# Ethical decision-making confidence among nurse leaders: a mixed-methods study of determinants and ethical implications

**DOI:** 10.3389/fmed.2026.1700102

**Published:** 2026-04-23

**Authors:** Huijing Shang, Lingfang Xiong, Kefan Chen, Renqiong Tian, Jing Tu, Xianhui Shang

**Affiliations:** 1 Department of Rheumatology and Immunology, The First People's Hospital of Zunyi (The Third Affiliated Hospital of Zunyi Medical University), Zunyi, China; 2 Department of Rheumatology and Immunology, The Second Affiliated Hospital of Guizhou University of Traditional Chinese Medicine, Guiyang, China; 3 Department of Pediatric Rehabilitation, The First People's Hospital of Zunyi (The Third Affiliated Hospital of Zunyi Medical University), Zunyi, China; 4 Department of Pediatric Surgery, Affiliated Hospital of Zunyi Medical University, Zunyi, China; 5 Department of Pediatric Surgery, Guizhou Children's Hospital, Zunyi, China

**Keywords:** ethical climate, ethical decision-making confidence, mixed-methods, moral resilience, nurse leaders

## Abstract

**Objective:**

In today’s complex healthcare environment, ethical decision-making confidence (EDMC) among nurse leaders plays a pivotal role in ensuring ethically sound nursing practice. While prior research has primarily emphasized ethical competence, empirical investigation of EDMC and its determinants among nurse leaders remains limited. This study aimed to assess the level of EDMC among nurse leaders in Guizhou Province and to identify its key influencing factors to inform targeted intervention strategies.

**Methods:**

A sequential explanatory mixed-methods design was adopted. In the quantitative phase, 611 nurse leaders from secondary and tertiary hospitals in Guizhou Province were recruited through convenience sampling. Data were collected using the Ethical Decision-Making Confidence Scale (EDMC), the Chinese version of the Rushton Moral Resilience Scale (RMRs), and the Hospital Ethical Climate Survey (HECS). Univariate analyses and stepwise multiple linear regression were performed to identify independent predictors of EDMC. In the qualitative phase, purposive sampling and semi-structured interviews were conducted to further explain quantitative findings, and data were analyzed using Colaizzi’s seven-step method.

**Results:**

Nurse leaders demonstrated overall moderate-to-high levels of EDMC, with nearly one-third reporting insufficient confidence. Moral resilience (*β* = 0.42, *p <* 0.001), hospital ethical climate (*β* = 0.35, *p <* 0.001), and professional title (*β* = 0.16, *p* = 0.021) were identified as significant predictors of EDMC. Qualitative analysis identified three interrelated domains influencing EDMC: individual factors (e.g., limited moral resilience and burnout), organizational factors (e.g., inadequate ethical climate and managerial support), and societal factors (e.g., resource constraints and public pressure).

**Conclusion:**

EDMC among nurse leaders is shaped by the interaction of individual psychological resources, organizational environments, and societal influences. Although confidence levels were generally high, notable vulnerability persists in ethically complex contexts. Interventions aimed at strengthening moral resilience, improving ethical climate, and providing structured ethics training may enhance ethical decision-making confidence. By focusing on ethical decision-making confidence rather than competence alone, this study offers empirically grounded insights for leadership development and ethics education.

## Introduction

With the rapid advancement of medical technology and the diversification of social and cultural values, ethical challenges in nursing practice have become increasingly complex and visible (1). Ethical decision-making refers to the process by which nurses confronted with moral conflicts analyze and select actions grounded in ethical principles and professional values, directly influencing patient rights, professional integrity, and care quality (2). Beyond cognitive and skills-based competence, increasing attention has been directed toward psychological factors that determine whether ethical judgments are enacted in practice (3). Within this context, ethical decision-making confidence (EDMC) has emerged as a relevant construct, defined as nurses’ confidence in making and implementing ethically appropriate decisions when facing dilemmas (4). Conceptually, EDMC can be understood as a domain-specific form of confidence closely aligned with self-efficacy theory, as it reflects belief in one’s capacity to carry out morally appropriate actions under uncertainty. Unlike generalized self-confidence, however, EDMC is explicitly anchored in professional ethical standards and value-based commitment, emphasizing readiness for moral action rather than perceived competence alone.

Nurse leaders play pivotal roles in organizational coordination, resource allocation, and clinical governance. Their ethical decision-making confidence shapes not only their own responses to moral challenges but also team climate, ethical norms, and patient outcomes at the organizational level (5). Evidence indicates that nurses with insufficient ethical confidence are more likely to experience moral distress—recognizing the ethically appropriate action yet feeling unable to implement it (6, 7). From a psychological perspective, low EDMC may weaken the translation of ethical judgment into action, thereby increasing vulnerability to moral distress, emotional exhaustion, and burnout. Persistent exposure to such conditions may undermine professional identity and elevate turnover intention; approximately 10–25% of nurse leaders reportedly leave their positions due to ethics-related distress (8–13), posing risks to workforce stability and care quality (14).

Research in nursing ethics has traditionally emphasized ethical competence, focusing on knowledge, moral reasoning, and judgment skills (2, 15). While such approaches address whether nurses can identify appropriate ethical options, they do not fully explain why ethically knowledgeable professionals may hesitate to act under organizational pressure or uncertainty. In contrast, the psychological dimension of ethical decision-making confidence has received comparatively limited empirical attention. Birkholz et al. (4) developed the Ethical Decision-Making Confidence Scale for nurse leaders, and Zhao et al. (16) validated its Chinese version in 2024. However, research has largely concentrated on measurement validation rather than examining determinants and contextual mechanisms. Emerging evidence suggests that moral resilience and ethical climate may influence ethical confidence (3, 17), yet theoretical integration remains limited. Linking EDMC with established constructs such as self-efficacy and moral distress offers a more coherent explanatory framework for understanding why competence does not automatically translate into ethical action.

Ethical competence and ethical decision-making confidence should therefore be conceptualized as related yet distinct components along a “competence–confidence–action” continuum. Ethical competence provides the cognitive foundation for judgment, whereas EDMC determines whether such competence is activated and sustained under moral conflict and organizational constraint (3, 17). Within this continuum, EDMC functions as a psychological mediator between knowledge and action, shaping trust in moral judgment and persistence in ethically appropriate behavior despite contextual pressures. Failure to distinguish these constructs may obscure critical mechanisms underlying ethical behavior, particularly among nurse leaders facing complex institutional demands.

Against this backdrop, the present study examined nurse leaders in hospitals across Guizhou Province using a mixed-methods design. Quantitative surveys assessed the level and determinants of EDMC, while qualitative interviews explored how individual, organizational, and societal factors shape ethical confidence in practice. By shifting the analytical focus from competence alone to ethical decision-making confidence, this study contributes to theoretical refinement in nursing ethics research and provides evidence to inform targeted leadership development and ethics education strategies.

## Methods

### Study design

This study adopted a sequential explanatory mixed-methods design, comprising both quantitative and qualitative components. The quantitative phase utilized a cross-sectional survey to evaluate the current status and influencing factors of ethical decision-making confidence (EDMC) among nurse leaders. The qualitative phase was conducted subsequently to provide contextual and experiential explanations for the quantitative findings, thereby enhancing the interpretability and explanatory depth of the results.

### Participants and sampling

#### Sampling strategy

In the quantitative phase, convenience sampling was employed based on feasibility considerations to recruit nurse leaders from tertiary and secondary hospitals in Guizhou Province who met the inclusion criteria. This approach was considered appropriate given the managerial roles, limited availability, and multi-center hospital settings of the target population, while ensuring an adequate sample size for statistical analysis.

In the qualitative phase, purposive sampling was adopted to select information-rich participants. Nurse leaders with diverse ages, professional titles, years of experience, and departmental backgrounds were intentionally recruited based on quantitative results, in order to capture varied perspectives and enhance the credibility of qualitative findings.

##### Inclusion criteria

1 At least 1 year of experience in a nursing leadership role;
2 Currently serving as a nursing director, head nurse, or specialist nurse with recognized leadership responsibilities;
3 Provided written informed consent and voluntarily participated in the study.

##### Exclusion criteria

1 Nurse leaders who were on extended leave or participating in off-site training or further study during the survey period;

2. Questionnaires with missing data ≥5% on key variables.

Nurse leaders who were unavailable during the survey period were excluded before questionnaire distribution and were therefore not included in the denominator used to calculate the response rate.

### Instruments

1 General Information Questionnaire: Developed by the research team based on a comprehensive literature review and study objectives, including demographic and occupational variables (age, gender, education, professional title, position, years of service, hospital type and grade, and department).
2 Ethical Decision-Making Confidence Scale (EDMC): Originally developed by Birkholz et al. (4) and translated and psychometrically validated in the Chinese context by Ne’eman et al. (3). The scale consists of 15 items across two dimensions—professional skills confidence (9 items) and behavioral confidence (6 items)—rated on a 5-point Likert scale. In the present study, the overall Cronbach’s *α* was 0.947, indicating excellent internal consistency.
3 Chinese Version of the Rushton Moral Resilience Scale (RMRs): Developed by Heinze et al. and translated by Yang et al. The scale includes four dimensions—response to moral adversity, moral efficacy, relational integrity, and personal integrity—comprising 16 items rated on a 4-point Likert scale. Total scores range from 16 to 64, with higher scores reflecting greater moral resilience. The Cronbach’s *α* in this study was 0.788, indicating acceptable reliability.
4 Hospital Ethical Climate Survey (HECS): Developed by Olson and translated into Chinese by Wang et al. The scale assesses ethical climate across five dimensions (relationships with nurses, patients, physicians, managers, and the hospital) using 25 items rated on a 5-point Likert scale. Higher scores indicate a more positive ethical climate. The Cronbach’s *α* in this sample was 0.964, demonstrating excellent reliability.

### Data collection

#### Quantitative phase

The formal survey was conducted between August 2024 and May 2025. All investigators received standardized training prior to data collection to ensure consistency in explanation and administration. Questionnaires were distributed only to nurse leaders who had been confirmed to meet the inclusion criteria, were present at the participating hospitals during the survey period, and agreed to participate after receiving an explanation of the study purpose. After obtaining informed consent, questionnaires were distributed face-to-face, completed anonymously, and collected immediately on-site. Participants completed the survey independently during non-peak working hours to minimize disruption and response bias. A total of 611 questionnaires were distributed and 611 were returned. The reported 100% response rate therefore referred specifically to eligible participants who were available during the survey period, agreed to participate, and accepted the questionnaire.

A pilot survey involving 30 nurse leaders was conducted prior to the formal investigation to assess clarity, feasibility, and completion time. Based on pilot feedback, minor revisions were made to questionnaire instructions and wording, ensuring content validity and participant comprehension.

#### Qualitative phase

Semi-structured interviews were conducted in quiet and private settings (e.g., department meeting rooms). Written informed consent was obtained prior to participation. All interviews were audio-recorded with participants’ permission, and relevant non-verbal cues were documented. Each interview lasted approximately 15–30 min and continued until thematic saturation was achieved.

The interview guide was developed based on quantitative findings and relevant literature, refined through expert consultation, and finalized following pilot testing, ensuring alignment with the study objectives.

#### Quality control

1 Preparation phase: Literature search strategies were systematically developed, and survey instruments and interview guides were refined through team discussions and pilot testing.
2 Data collection phase: Investigators were uniformly trained, and standardized explanations regarding study purpose, anonymity, and voluntariness were provided to minimize interviewer bias and enhance data authenticity.
3 Data processing phase: Quantitative data were independently entered and cross-checked by two researchers. Qualitative interview data were transcribed verbatim within 24 h and independently coded by two researchers.
4 Analysis phase: Quantitative data underwent logical checks and missing-value handling prior to analysis. Qualitative data were analyzed using Colaizzi’s seven-step method, with member checking conducted to enhance trustworthiness and credibility.

#### Data analysis

Quantitative data were processed using Microsoft Excel 2019 and analyzed with SPSS version 29.0. Categorical variables were expressed as frequencies and percentages, while continuous variables were described as means ± standard deviations or medians with interquartile ranges, depending on normality.

Univariate analyses were conducted with EDMC scores as the dependent variable, followed by stepwise multiple linear regression analysis to identify influencing factors. Prior to regression analysis, key statistical assumptions—including multicollinearity, residual normality, and homoscedasticity—were examined and met, supporting the appropriateness of the regression model. All statistical tests were two-tailed, with *p <* 0.05 considered statistically significant.

Qualitative data were analyzed using Colaizzi’s seven-step method, with themes and subthemes extracted. Independent coding by two researchers ensured analytical rigor, with discrepancies resolved through discussion and consensus.

### Ethical considerations

This study was approved by the Ethics Committee of the First People’s Hospital of Zunyi. Written informed consent was obtained from all participants. Participation was entirely voluntary, and confidentiality and anonymity were strictly maintained. Data were anonymized and used exclusively for research purposes. In qualitative interviews, participant identities were replaced with codes, and audio recordings were accessible only to the research team and securely stored upon study completion.

## Results

### General characteristics

A total of 611 questionnaires were distributed to eligible nurse leaders who were available during the survey period and agreed to participate. All 611 questionnaires were returned and met the predefined data quality criteria, yielding a valid response rate of 100% among those who received the questionnaire. The majority of participants were female (94.8%). Most participants were aged 25–34 years (48.0%) or 35–44 years (41.2%), and the predominant educational background was a bachelor’s degree (90.7%). These characteristics reflect the typical demographic composition of nurse leaders in secondary and tertiary hospitals in the study region. Detailed participant characteristics are presented in Table 1.

**Table 1:** General characteristics of participants (*n =* 611).

Characteristic	Category	*n*	%
Gender	Female	579	94.8
	Male	32	5.2
Age (years)	<25	32	5.2
	25–34	293	48.0
	35–44	252	41.2
	≥45	34	5.6
Education	Diploma	52	8.5
	Bachelor	554	90.7
	Graduate	5	0.8

### Levels of ethical decision-making confidence

The mean total score on the Ethical Decision-Making Confidence Scale (EDMC) was 37.03 ± 8.71, with an interquartile range of 30.00–41.50, indicating an overall moderate-to-high level of ethical decision-making confidence among nurse leaders. The mean scores for the professional skills confidence and behavioral confidence dimensions were 18.50 ± 4.39 and 18.53 ± 4.41, respectively.

**Figure 1:**
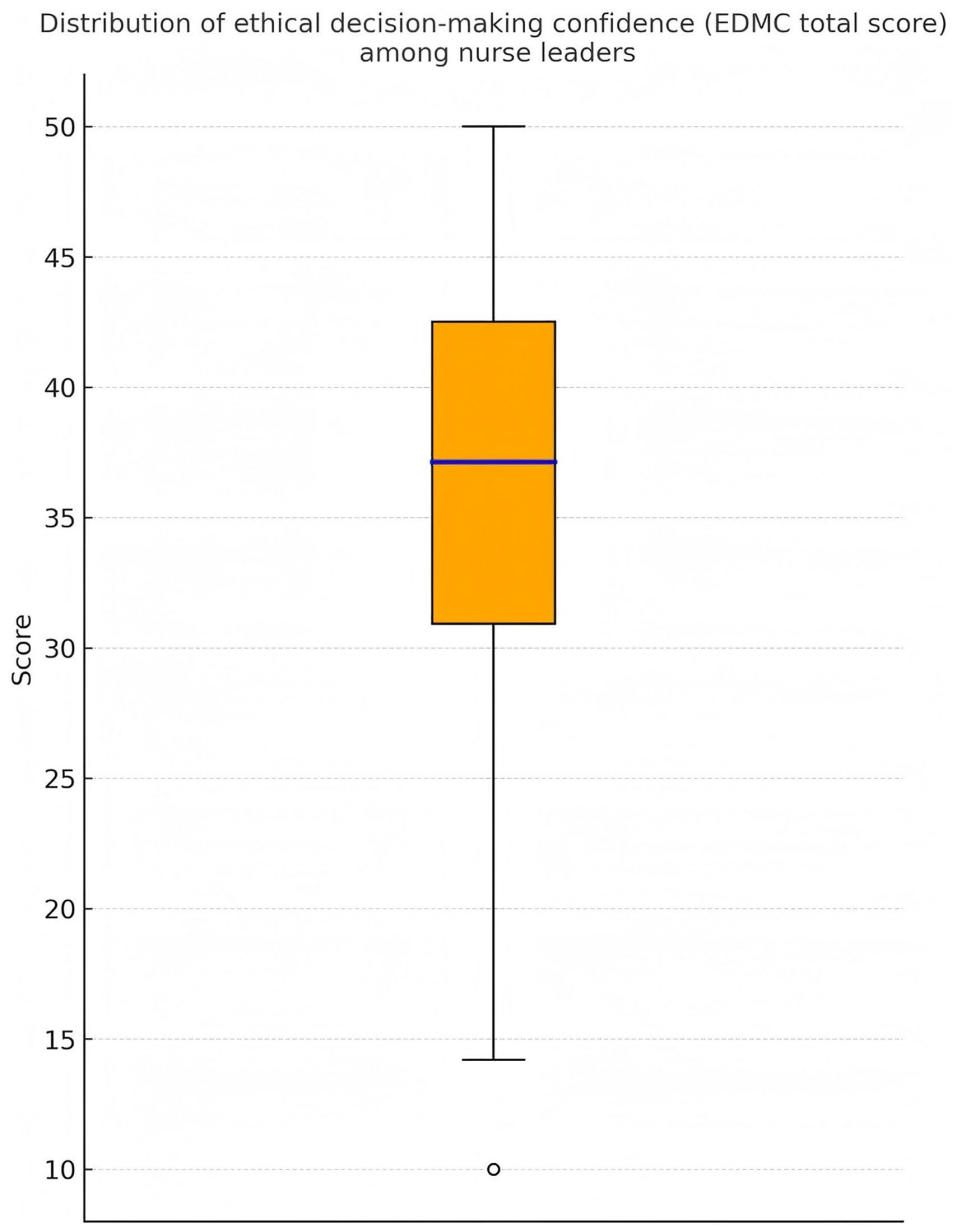
Distribution of ethical decision-making confidence (EDMC total score) among nurse leaders. The boxplot shows that confidence was generally at a moderate-to-high level, with a small number of participants scoring notably lower.

Despite the relatively high overall confidence level, score dispersion indicated notable individual variability, with a subset of participants exhibiting comparatively low confidence levels. Specifically, nearly one-third of nurse leaders scored at the lower end of the distribution, suggesting the presence of a potentially vulnerable subgroup. This variability highlights that, although average confidence appears adequate, a considerable proportion of leaders may experience hesitation or reduced decisional assurance in ethically complex situations. The distribution of EDMC total scores is illustrated in Figure 1, and detailed descriptive statistics are shown in Table 2.

**Table 2:** Scores of ethical decision-making confidence among nurse leaders (*n =* 611).

Dimension	*n*	Mean ± SD	Min	P25	Median	P75	Max
Total score	611	37.03 ± 8.71	10.0	30.0	38.0	41.5	50.0
Professional skills confidence	611	18.50 ± 4.39	5.0	15.0	19.0	21.0	25.0
Behavioral confidence	611	18.53 ± 4.41	5.0	15.0	19.0	21.0	25.0

The visible lower-tail dispersion further supports the existence of a subgroup with comparatively limited confidence, warranting attention from nursing management.

### Comparisons of ethical decision-making confidence across participant characteristics

Univariate analyses revealed statistically significant differences in EDMC scores across age groups (*F* = 3.94, *p* = 0.008). Participants aged 25–34 years reported the highest confidence scores (38.19 ± 8.57), whereas those aged 35–44 years reported the lowest scores (35.64 ± 8.82).

No statistically significant differences in EDMC scores were observed by gender (*p* = 0.671) or educational background (*p* = 0.073). However, a descriptive trend was noted in which participants with diploma-level education reported higher mean confidence scores than those with bachelor’s or graduate degrees, although this difference did not reach statistical significance. Due to the graduate-level subgroup comprising only five participants, results related to educational background should be interpreted with caution and were not given substantive inferential weight in subsequent analyses. Results of univariate analyses are summarized in Table 3.

**Table 3:** Comparison of ethical decision-making confidence across demographic characteristics (*n =* 611).

Characteristic	Category	*n*	Mean	SD	Test	Statistic	*p-*value
Gender	Female	579	37.07	8.73	*t*-test	−0.43	0.671
	Male	32	36.41	8.47			
Age (years)	<25	32	37.28	8.18	ANOVA	3.94	0.008
	25–34	293	38.19	8.57			
	35–44	252	35.64	8.82			
	≥45	34	37.06	8.45			
Education	Diploma	52	38.44	8.44	ANOVA	2.63	0.073
	Bachelor	554	36.97	8.74			
	Graduate	5	29.40	1.95			

### Factors influencing ethical decision-making confidence

Variables that demonstrated statistical significance in univariate analyses were entered into a stepwise multiple linear regression model. Regression diagnostics confirmed that key assumptions were met, and the final model was statistically significant.

In response to the extremely small graduate-level subgroup, educational level was not retained as a reportable predictor. The final model identified moral resilience, hospital ethical climate, and professional title as significant factors associated with ethical decision-making confidence. The regression results are presented in Table 4.

**Table 4:** Stepwise multiple linear regression analysis of ethical decision-making confidence among nurse leaders.

Variable	*β*	SE	Standardized *β*	*t*	*P-*value
Constant	21.34	3.28	–	6.50	<0.001
Moral resilience	0.42	0.06	0.39	7.00	<0.001
Hospital ethical climate	0.35	0.07	0.33	5.11	<0.001
Professional title	0.16	0.07	0.12	2.32	0.021

Educational attainment was excluded from the final regression model because the graduate-level category included only five participants (*n* = 5), limiting the statistical stability and interpretability of this variable.

### Qualitative findings

Qualitative analysis of interviews with 21 nurse leaders, conducted using Colaizzi’s seven-step method, yielded three overarching thematic domains that influenced ethical decision-making confidence:

1 Individual-level factors, including insufficient moral resilience, increased job burnout, and inadequate ethical knowledge;
2 Organizational-level factors, including poor ethical climate, limited managerial support, and lack of accessible ethical resources;
3 Societal-level factors, including conflicts between patient or family expectations and available healthcare resources, as well as pressure from public opinion

Representative quotations illustrating each theme are presented in Table 5, demonstrating how ethical decision-making confidence is shaped by the interaction of personal, organizational, and societal influences. These qualitative findings further contextualize the quantitative dispersion, suggesting that lower confidence scores may reflect cumulative psychological and organizational pressures rather than isolated individual deficiencies.

**Table 5:** Themes and representative quotations from qualitative interviews.

Theme level	Subtheme	Representative quotation
Individual	Insufficient moral resilience	“I know what is right, but often I lack the courage to persist.”
Organizational	Lack of managerial support	“When facing ethical dilemmas, I hope to receive support from the nursing administration, but often I must endure it alone.”
Societal	External pressures	“Questions from families and the media make me doubt whether my judgment is correct.”

## Discussion

### Key findings

This mixed-methods study is the first to systematically examine ethical decision-making confidence (EDMC) as a distinct construct among nurse leaders in Guizhou Province. The findings revealed that although overall EDMC levels were moderate to high, nearly one-third of nurse leaders demonstrated insufficient confidence, indicating a non-negligible vulnerability when facing ethically complex situations. This dispersion suggests that average confidence levels may mask a clinically and managerially meaningful subgroup at risk of decisional hesitation, increased moral distress, and potential leadership instability. Quantitative analyses identified moral resilience, hospital ethical climate, and professional title as significant determinants of EDMC, while qualitative interviews further elucidated how individual psychological resources, organizational support, and sociocultural pressures jointly shape ethical confidence in real-world practice. By integrating quantitative and qualitative evidence, this study moves beyond descriptive assessment and offers a multidimensional explanation of ethical decision-making confidence among nurse leaders.

### Comparison with previous research

Most existing research in nursing ethics has focused on ethical decision-making ability or ethical competence among clinical nurses, with comparatively limited attention given to nurse leaders (2, 15). Competence-oriented studies primarily address ethical knowledge and reasoning skills but often overlook the psychological processes that influence whether ethical judgments are confidently enacted in practice. Consistent with prior evidence, the present study found that although nurse leaders generally reported higher confidence levels than frontline nurses, a substantial proportion still exhibited inadequate EDMC, suggesting that leadership status alone does not eliminate ethical vulnerability. Importantly, this finding highlights that formal authority does not necessarily translate into psychological readiness for ethical action under pressure.

The positive association between moral resilience and EDMC observed in this study is consistent with previous findings highlighting the protective role of psychological resilience against moral distress and ethical uncertainty (3, 8). Recent empirical studies have further demonstrated that moral integrity and moral self-concept contribute to nurses’ ethical sensitivity and confidence, reinforcing the centrality of internal psychological resources in ethical decision-making processes (3). In addition, the strong predictive effect of hospital ethical climate aligns with prior research linking supportive ethical environments to reduced moral distress, burnout, and turnover intention among nurses (7, 12, 18). Recent evidence among nurse managers further confirms that ethical climate is closely associated with leadership stability and ethical decision outcomes, particularly in high-pressure clinical settings (14). Moreover, ethical leadership has been shown to foster organizational citizenship behaviors and mitigate ethical conflict by shaping a supportive and value-consistent work environment (19). Accordingly, our findings further support the importance of strengthening both individual psychological resources and organizational ethical environments to enhance EDMC among nurse leaders.

### Mechanistic explanations

Ethical decision-making confidence may be conceptualized as a psychological bridge between ethical competence and ethical action. While ethical competence enables nurse leaders to identify ethically appropriate options, EDMC determines whether they trust their judgment and feel sufficiently prepared to act under conditions of uncertainty, moral conflict, and organizational constraint. From a theoretical perspective, EDMC shares conceptual proximity with domain-specific self-efficacy, as it reflects belief in one’s capability to enact morally appropriate behavior; however, it is distinguished by its explicit grounding in professional ethical standards and value-based commitment rather than generalized personal confidence. Nurse leaders with high moral resilience are better able to maintain value integrity and emotional stability when confronted with ethical adversity, thereby sustaining confidence in decision-making (9–11). A positive hospital ethical climate further reinforces this process by providing institutional support, shared ethical norms, and opportunities for open ethical dialogue.

Conversely, inadequate organizational support and heightened public scrutiny may exacerbate moral distress and erode confidence, even among experienced leaders. In this sense, insufficient EDMC may function as a psychological vulnerability factor that amplifies the impact of moral distress by weakening the translation of ethical judgment into ethical action. Such vulnerability may contribute to delayed decisions, avoidance behaviors, or over-reliance on hierarchical authority, thereby potentially compromising timely and ethically grounded leadership responses. Qualitative findings from this study illustrate that insufficient ethical knowledge, emotional exhaustion, limited managerial backing, and sociocultural pressure often converge to undermine EDMC, rather than acting as isolated influences. Recent literature conceptualizes moral resilience as a dynamic capacity that can be strengthened through organizational and educational interventions, supporting its role as a key mechanism underlying ethical confidence (17, 20, 21). Within this framework, moral resilience may operate as a buffering mechanism that stabilizes ethical self-efficacy and protects confidence under morally adverse conditions. Furthermore, fostering moral courage has been recognized as a complementary process through which nurse leaders can sustain ethical confidence when institutional or external constraints limit ethical action (22).

### Implications for practice and management

The findings of this study have direct and actionable implications for nursing management and leadership development. First, ethics-related education should move beyond traditional knowledge-based instruction to incorporate case-based ethics training, simulation-based ethical decision-making exercises, and reflective discussion, particularly for nurse leaders who demonstrate lower EDMC or have less leadership experience. Given that nearly one-third of leaders demonstrated insufficient confidence, early identification of low-confidence subgroups and provision of structured support should be prioritized within leadership development programs. Second, healthcare institutions should prioritize cultivating supportive ethical climates by establishing ethics consultation teams, regular interdisciplinary ethics forums, and accessible ethical support pathways for nurse leaders.

Third, moral resilience should be actively strengthened through mental health support services, stress management programs, and structured peer support networks, enabling nurse leaders to cope more effectively with ethical challenges (20, 21). Finally, given the growing impact of public opinion and media scrutiny, hospitals and professional organizations should implement clear communication strategies and crisis-response mechanisms to mitigate confidence erosion during ethically sensitive events. Cross-cultural evidence suggests that such organizational and educational interventions are both feasible and effective across diverse healthcare systems, underscoring their broad applicability (23).

### Limitations and future directions

Several limitations should be acknowledged. First, the cross-sectional design precludes causal inference; therefore, longitudinal and intervention-based studies are needed to clarify causal pathways and to examine changes in ethical decision-making confidence (EDMC) over time. Second, participants were recruited exclusively from hospitals in Guizhou Province, a region characterized by specific healthcare resource distribution patterns, organizational structures, and sociocultural contexts. These contextual characteristics may differ from those of more economically developed regions or other national healthcare systems, potentially limiting the external validity and international generalizability of the findings. Although core determinants identified in this study—such as moral resilience and hospital ethical climate—have demonstrated relevance in cross-cultural nursing research, their magnitude and underlying mechanisms may vary across institutional and cultural environments. Therefore, caution is warranted when extrapolating these findings beyond the study setting. Third, although thematic saturation was achieved, the relatively small qualitative sample may not fully capture the diversity of experiences among nurse leaders. In addition, the extremely small number of participants with graduate-level education (*n =* 5) limited the stability and interpretability of education-related comparisons; therefore, educational attainment was not treated as a robust explanatory factor in this study.

Future research should adopt multicenter, large-scale, and cross-regional or cross-national mixed-methods designs to examine the structural stability and contextual variability of EDMC across diverse healthcare systems. Longitudinal investigations are needed to explore the dynamic development of ethical confidence, and rigorously designed intervention studies should evaluate the effectiveness of targeted organizational and educational strategies. Furthermore, the development, cultural adaptation, and empirical validation of context-specific ethics training and institutional support programs will be essential to advance evidence-based approaches for strengthening nurse leaders’ ethical decision-making confidence in varied healthcare environments. Future studies may also consider stratified sampling strategies to ensure adequate representation across educational levels and to enhance statistical power in subgroup analyses.

## Revised conclusion

In conclusion, this study demonstrates that nurse leaders’ ethical decision-making confidence is a multidimensional construct shaped by the interaction of individual psychological resources, organizational ethical environments, and broader sociocultural pressures. Although overall EDMC levels were moderate to high, significant gaps remain, particularly in ethically complex and high-pressure contexts. The presence of a substantial low-confidence subgroup underscores the need for proactive organizational strategies to prevent ethical hesitation and leadership strain. Moral resilience, hospital ethical climate, and professional title emerged as key determinants of confidence.

By explicitly shifting the analytical focus from ethical competence to ethical decision-making confidence and integrating quantitative and qualitative evidence, this study advances existing nursing ethics and leadership research. The findings provide a robust foundation for designing multifaceted, practice-oriented interventions aimed at strengthening ethical confidence, promoting ethical leadership, and safeguarding patient rights. From a broader medical ethics perspective, enhancing ethical decision-making confidence empowers nurse leaders to translate ethical judgment into action, reinforces professional integrity, and supports the development of ethically responsible healthcare institutions.

## Data Availability

The original contributions presented in the study are included in the article/supplementary material, further inquiries can be directed to the corresponding author.
